# Supplementary material for: The effect of a task-specific training on upper limb performance and kinematics while performing a reaching task in a fatigued state
Source: PLoS One. 2024 Jan 22;19(1):e0297283. doi: 10.1371/journal.pone.0297283 (PMC10802943; doi:10.1371/journal.pone.0297283)
Supplement: S1 Fig — Top left: positions of the targets relative to the participant (Target 1 = 90° of humeral abduction and 90° of external rotation, elbow flexed at 90°, Target 2 = 90° of shoulder abduction, elbow extended, Target 3 = 120° of shoulder scaption, elbow extended, Target 4 = 120° of shoulder flexion, elbow extended and Target 5 = 140° of shoulder flexion, elbow extended. Bottom left: vision of the participant in the virtual reality environment. Right: A left-handed participant in initial position. (DOCX) [file pone.0297283.s001.docx]

**
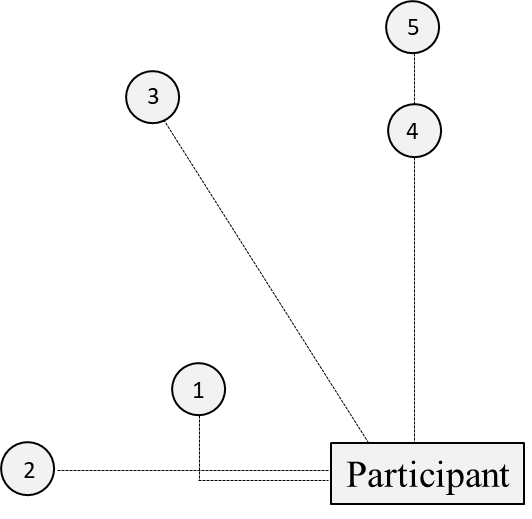
Figure S1. Experimental setup**

**
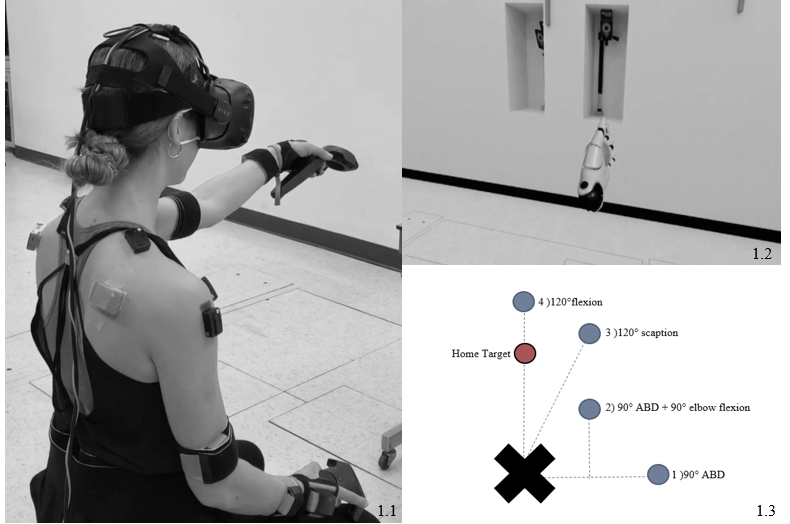
**

**
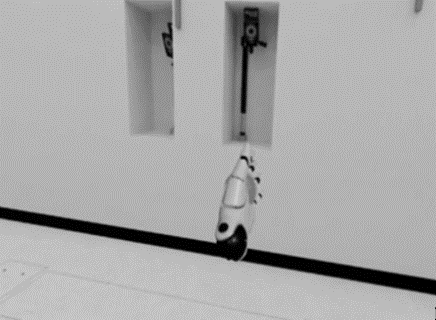
**

**Experimental setup.** Top left: positions of the targets relative to the participant (Target 1= 90° of humeral abduction and 90° of external rotation, elbow flexed at 90°, Target 2= 90° of shoulder abduction, elbow extended, Target 3= 120° of shoulder scaption, elbow extended, Target 4= 120° of shoulder flexion, elbow extended and Target 5= 140° of shoulder flexion, elbow extended. Bottom left: vision of the participant in the virtual reality environment. Right: A left-handed participant in initial position.
