# Supplementary material for: The effect of a task-specific training on upper limb performance and kinematics while performing a reaching task in a fatigued state
Source: PLoS One. 2024 Jan 22;19(1):e0297283. doi: 10.1371/journal.pone.0297283 (PMC10802943; doi:10.1371/journal.pone.0297283)
Supplement: Attachment — Submitted filename: Response to reviewer-11dec2023.docx [file pone.0297283.s003.docx]

Reviewer #1: Overall comments. The introduction has undergone significant enhancement. The study's objective has been explicitly articulated. The discussion is now more focused on the results and has been augmented with pertinent information. I would like to access the supplementary material of the article to view the explanatory images related to the exercises conducted in the study. Additionally, I have observed that the version of the manuscript with Track Changes does not precisely match the one without Track Changes. Some sentences in the text still require changes.

Lines 84-85: I suggest re-formulating this in a more scientific language.

- Done

Lines 86-88: I suggest adding a reference here.

- Done, the reference added summarizes the current state of knowledge on fatigue.

Lines 88-90: The sentence is repetitive. You may remove “that reduce the occurrence of such changes in a fatigued state” (lines 89-90).

- Removed as suggested.

Lines 98-99: I suggest adding a reference here.

- References 12 to 14 should have been placed after this sentence; consequently, they were relocated accordingly.

Lines 129-133: I do think it is necessary to express angles using joint anatomical planes, in this case referred to the shoulder or elbow, avoiding expression such as humeral abduction and rotation (Target 1), shoulder scaption (Target 3) by expressing them as combination of sagittal and frontal plane movements.

- The planes of movement were added as suggested. However, we believe that some readers would prefer the anatomical angles, so we decided to keep this information within brackets.

Line 133: Unfortunately, I cannot see the supplementary file.

- We don’t know why the supplementary file is not available as it has been submitted. Here is the Figure S1:

**Figure S1. Experimental setup**

**Experimental setup.** Top left: positions of the targets relative to the participant (Target 1= 90° of humeral abduction and 90° of external rotation, elbow flexed at 90°, Target 2= 90° of shoulder abduction, elbow extended, Target 3= 120° of shoulder scaption, elbow extended, Target 4= 120° of shoulder flexion, elbow extended and Target 5= 140° of shoulder flexion, elbow extended. Bottom left: vision of the participant in the virtual reality environment. Right: A left-handed participant in initial position.

Line 136-138: The description of the starting point should be improved. The sentence is not clear and fluent, more confusing than explanatory. The verb “to reach” is repetitive.

- We made some modifications to improve the description as follow:
- *‘’ To standardize this starting position, an additional target (5cm radius ball) was positioned in front of the participant, at 90° of humeral elevation (in the sagittal plane, elbow extended). Participants were required to return to this target between each reaching movement to initiate the release of the subsequent target**.’’*

Lines 153: Is the closing parenthesis at the end of the sentence an error?

- Yes, it is, thank you for noticing.

Lines 153-154: I think it is unnecessary to express dumbbell weight in pound, as measurement units must be expressed according to the International System of Units guidelines.

- We agree, we removed this additional information.
